# Nomogram-based prediction model for tocilizumab-induced leukopenia in adults using FAERS and PMDA data

**DOI:** 10.1590/1414-431X2026e15395

**Published:** 2026-08-21

**Authors:** Lujing Wang, Xiaochun Zhang, Xinglan Bao, Yan Chen, Hongjian Yuan

**Affiliations:** 1 Department of Rheumatology, Taizhou Second People’s Hospital Affiliated to Yangzhou University, Taizhou, Jiangsu, China; 2 Department of Colorectal Surgery, The Second Affiliated Hospital of Nanjing University of Chinese Medicine, Nanjing, Jiangsu, China

**Keywords:** Tocilizumab, Leukopenia, FAERS, PMDA, Prediction model, Nomogram

## Abstract

Tocilizumab, an interleukin-6 receptor antagonist used for immune-mediated diseases, is associated with leukopenia, a serious adverse event that increases infection risk. However, no predictive model exists to identify adult patients at high risk for this complication. The aim of this study was to develop and validate a clinical prediction model for tocilizumab-induced leukopenia in adults using real-world pharmacovigilance data. This observational study utilized data from the FDA Adverse Event Reporting System (FAERS) for model development (internal training/test sets; n=5,059) and the Japanese Pharmaceuticals and Medical Devices Agency (PMDA) database for external validation (n=1,338). A nomogram was developed using multivariate logistic regression to identify key predictive factors. Multivariable analysis identified severe COVID-19 (P<0.001), higher tocilizumab dose (P=0.011), younger age (P<0.001), and lower body weight (P<0.001) as independent predictors of leukopenia. The nomogram demonstrated good discriminative ability, with area under the curve (AUC) values of 0.709 (internal training), 0.792 (internal test), and 0.761 (external validation). Calibration and decision curve analysis confirmed the model's robustness and clinical utility. Risk stratification effectively identified high-risk patients. This novel nomogram provides a practical, evidence-based tool for predicting tocilizumab-induced leukopenia in adults. It can assist clinicians in identifying high-risk patients for closer monitoring and personalized management, potentially improving treatment safety.

## Introduction

**Figure 1:**
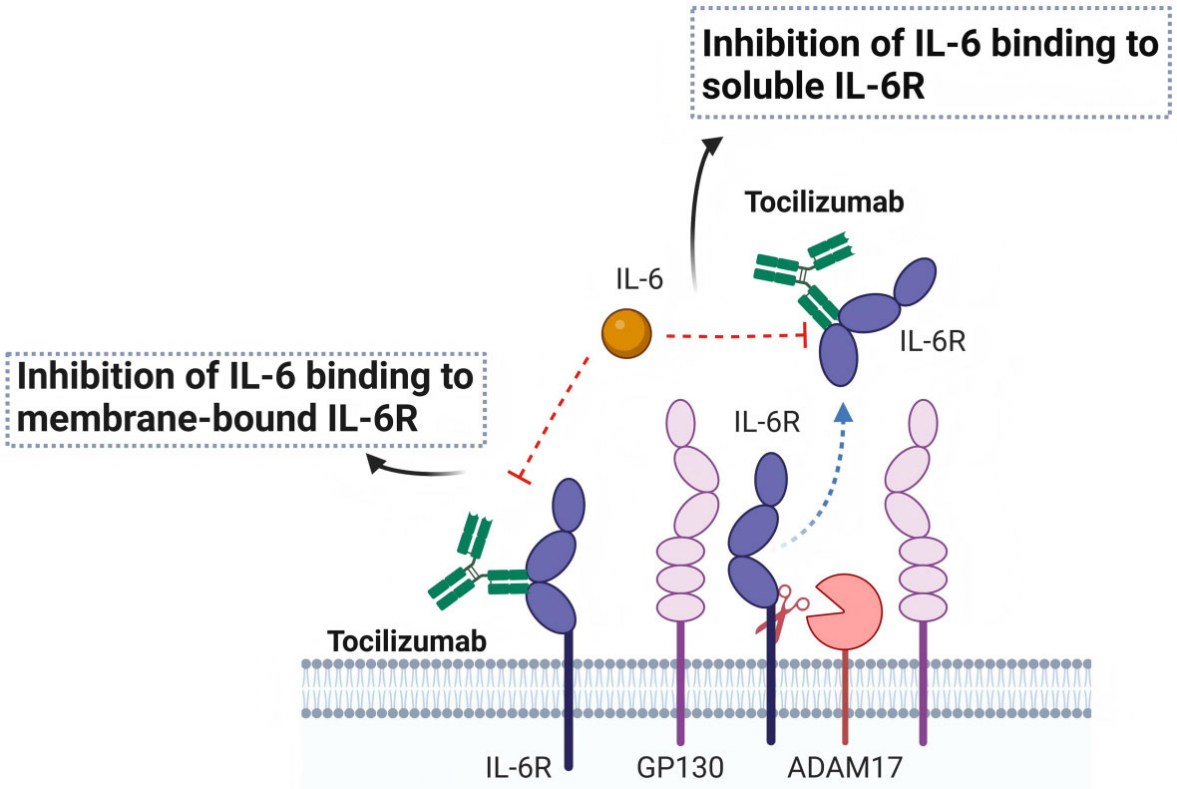
Tocilizumab inhibits interleukin-6 (IL-6) signaling by binding to the IL-6 receptor (IL-6R) through two mechanisms.

Tocilizumab, a monoclonal antibody targeting the interleukin-6 (IL-6) receptor, is widely used in the treatment of immune-mediated diseases such as rheumatoid arthritis, systemic juvenile idiopathic arthritis, and giant cell arteritis (1-3). By inhibiting the IL-6 signaling pathway, tocilizumab reduces inflammatory responses and improves clinical outcomes. This process is depicted in Figure 1. However, as an immunosuppressive agent, tocilizumab is associated with adverse effects, including leukopenia, which can increase the risk of infections and complicate patient management in adults (4).

Leukopenia, characterized by a reduction in peripheral white blood cell (WBC) count, is a significant concern during tocilizumab therapy. The mechanism underlying tocilizumab-induced leukopenia is not fully understood but is thought to involve the suppression of IL-6 signaling, which plays a critical role in hematopoiesis and immune cell regulation (5,6). In adult populations, leukopenia is particularly concerning due to age-related declines in bone marrow reserve and comorbidities (7). Early identification of high-risk adults is critical to optimizing therapeutic safety.

Despite the clinical importance of leukopenia, there is a lack of predictive models to assess the risk of this adverse event in adult patients receiving tocilizumab. Recent advances in pharmacovigilance and the availability of large-scale databases, such as the Food and Drug Administration (FDA) Adverse Event Reporting System (FAERS) and the Japanese Pharmaceuticals and Medical Devices Agency (PMDA) database, provide an opportunity to develop predictive models using real-world data (7-9). However, no such models have been developed specifically for leukopenia in tocilizumab-treated patients.

This study aimed to develop and validate a clinical prediction model for leukopenia in adult patients receiving tocilizumab, leveraging real-world data from the FAERS and the PMDA databases. By identifying key predictive factors - such as COVID-19, dosage, age, and weight - we sought to create a nomogram that enables clinicians to stratify patients based on their risk of developing leukopenia. The primary objective was to provide a practical, evidence-based tool for early identification of high-risk individuals, facilitating personalized treatment strategies and improving the safety and efficacy of tocilizumab therapy. This model represents a significant step toward precision medicine in the management of immune-mediated diseases.

## Material and Methods

### Study population

This observational study used data from the FAERS and PMDA databases. These databases were chosen due to their large scale, international scope, standardized reporting, and specific inclusion of adverse events related to tocilizumab. We identified reports where tocilizumab was the primary suspected drug, spanning the period from January 1, 2010 to September 30, 2024. This timeframe was selected to capture post-approval safety data following its initial authorization for rheumatoid arthritis by the US FDA (2009) and Japan PMDA (2008). The 2010 start date ensured the inclusion of real-world evidence from the phase of widespread clinical adoption, allowing robust assessment of long-term safety profiles. To ensure consistency in identifying adverse events such as leukopenia and neutropenia, we developed and adhered to a dedicated study manual (Supplementary Material S1). First, relevant preferred terms (PTs) from MedDRA (version 26.1) were selected as candidate terms, and the terminology set was preliminarily screened and grouped according to MedDRA term selection criteria and the Standardized MedDRA Queries (SMQ) guidelines. Two independent reviewers with expertise in adverse drug reaction identification performed blinded manual mapping and verification of PTs or Lowest Level Terms (LLTs) from FAERS and PMDA reports. In cases of disagreement, a third adjudicator reviewed the records and documented the final decision along with the rationale. A complete list of PTs, their corresponding LLTs, and mapping annotations is provided in Supplementary Material S1. This process was conducted in accordance with the official MedDRA guidelines and the recommended practices for SMQ implementation (10). Duplicate entries (e.g., same patient ID, event, and timeline) were identified and merged using FDA-recommended deduplication algorithms. FAERS and PMDA data undergo preliminary validation by regulatory agencies (11). Further subjective “cleaning” risks introducing investigator bias. Instead, we prioritized transparency by retaining raw data structures while applying robust statistical methods to mitigate noise.

### Inclusion and exclusion criteria

The dataset included all FAERS and PMDA reports where tocilizumab was identified as the primary suspected drug. Exclusion criteria were: 1) patients aged <19 years (to focus on adults and avoid pediatric pharmacokinetic variability); 2) individuals with body weight <15 or >150 kg; 3) cases with missing critical variables (e.g., age, weight, dosage, leukopenia status); and 4) reports containing evidence of viral infection.

Leukopenia is defined as a sustained white blood cell count of less than 4.0×10^9^/L in peripheral blood. The FAERS database and the corresponding fields in the PMDA database have been used to identify cases of leukopenia, without specific diagnostic criteria being required.

**Figure 2:**
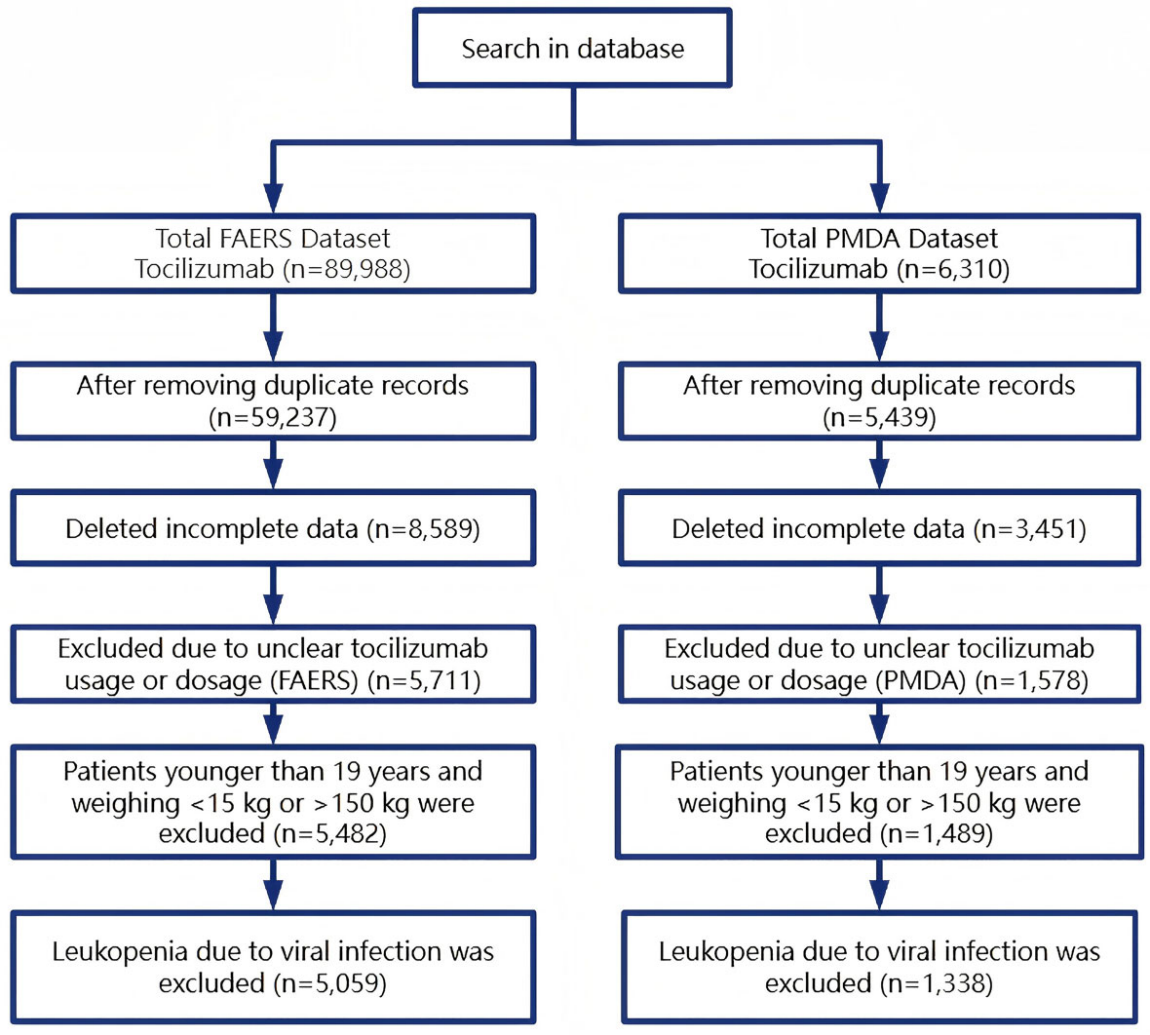
Data screening and inclusion process for the FDA Adverse Event Reporting System (FAERS) and Pharmaceuticals and Medical Devices Agency (PMDA) databases.

The data filtering process resulted in a dataset consisting of 5,059 patients from the FAERS database and a cohort of 1,338 patients from the PMDA database (Figure 2).

### Model construction and statistical analysis

The FAERS dataset was divided into an internal training set (70%) and an internal test set (30%) using stratified random sampling based on the outcome variable (leukopenia status) to ensure proportional representation of cases and controls in both groups. This approach minimizes selection bias and maintains balanced class distribution. The PMDA dataset served as an external test set to validate generalizability. The PMDA participants served as the external testing group. Data analysis was performed using R 4.2.2 and SPSS 26.0 software (IBM, USA). The Shapiro-Wilk test was used to assess the normality of continuous variables. Normally distributed data are reported as means±SD, while non-normally distributed data are represented as median and interquartile range. For normally distributed data, Student's *t*-test was employed. For skewed distributions, the Wilcoxon-Mann-Whitney U test was utilized. Categorical variables were described using frequency (percentage), and comparisons were made using Pearson's chi-squared test or Fisher's exact test, as appropriate. Pre-screening data were normalized. To mitigate the risk of type I errors due to multiple hypothesis testing, we applied the Benjamini-Hochberg procedure (false discovery rate [FDR] correction) during variable selection in univariate analysis. Spearman correlation and Belsley collinearity tests were used to assess collinearity across all covariables. The prediction model was developed using multivariate stepwise backward logistic regression analysis, and the nomogram was constructed using the “rms” package. Receiver operating characteristic (ROC) curves were generated to evaluate the predictive performance of the model, and the area under the curve (AUC) was calculated through ROC curve analysis. Decision curve analysis (DCA) and clinical impact curve analysis (CICA) were used to validate the clinical applicability of the nomogram. Finally, risk factors were stratified by scoring each independent factor in the nomogram. A significance level of P<0.05 was considered statistically significant.

This study was conducted in accordance with the ethical principles outlined in the Declaration of Helsinki. The use of de-identified publicly available data did not require approval from an institutional review board or informed consent.

## Results

### Demographic and clinical characteristics of the study population

A total of 6,397 patients from three cohorts were analyzed: the internal training set (N=3,561), the internal testing set (N=1,498), and the external testing set (N=1,338). Baseline demographic and clinical characteristics of the 6,397 patients are summarized in Table 1. Overall, rheumatoid arthritis was the predominant indication (4,980/6,397; 77.90%), while Castleman's disease (1.41%), Still's disease (1.55%), Takayasu's arteritis (0.81%), and juvenile idiopathic arthritis (0.41%) were uncommon. The prevalence of COVID-19 and giant cell arteritis in the entire cohort was 7.22 and 11.24%, respectively. To assess the potential impact of COVID-19 severity on tocilizumab-associated leukopenia, we performed a stratified analysis classifying patients into three groups. Based on available clinical severity indicators (e.g., hospitalization, ICU admission, mechanical ventilation, or death), patients with COVID-19 were further categorized as severe (1.02%) or mild (6.21%). Intravenous infusion was the most frequent administration route, with subcutaneous injection used in 28.9%. The population was predominantly female (4,698/6,397; 73.40%). The overall incidence of leukopenia was 2.50% (160/6,397) and did not differ significantly among the internal training (2.78%), internal test (1.94%), and external test (2.39%) cohorts (P=0.206).

**Table 1:** Demographic and baseline characteristics of patients.

Characteristic	All patients (n=6397)	Internal training set (n=3561)	Internal test set (n=1498)	External test set (n=1338)	P-value^*^
Castleman's Disease, n (%)					0.607
No	6307 (98.59)	3511 (98.60)	1480 (98.80)	1316 (98.36)	
Yes	90 (1.41)	50 (1.40)	18 (1.20)	22 (1.64)	
COVID-19, n (%)					0.739
No	5935 (92.78)	3307 (92.87)	1382 (92.26)	1246 (93.12)	
Mild	397 (6.21)	215 (6.04)	100 (6.68)	82 (6.13)	
Severe	65 (1.02)	39 (1.10)	16 (1.07)	10 (0.75)	
Giant Cell Arteritis, n (%)					0.570
No	5678 (88.76)	3149 (88.43)	1340 (89.45)	1189 (88.86)	
Yes	719 (11.24)	412 (11.57)	158 (10.55)	149 (11.14)	
Juvenile Idiopathic Arthritis, n (%)					0.822
No	6371 (99.59)	3545 (99.55)	1493 (99.67)	1333 (99.63)	
Yes	26 (0.41)	16 (0.45)	5 (0.33)	5 (0.37)	
Rheumatoid Arthritis, n (%)					0.905
No	1417 (22.15)	796 (22.35)	327 (21.83)	294 (21.97)	
Yes	4980 (77.85)	2765 (77.65)	1171 (78.17)	1044 (78.03)	
Still's Disease, n (%)					0.581
No	6298 (98.45)	3511 (98.60)	1472 (98.26)	1315 (98.28)	
Yes	99 (1.55)	50 (1.40)	26 (1.74)	23 (1.72)	
Takayasu's Arteritis, n (%)					0.957
No	6345 (99.19)	3533 (99.21)	1485 (99.13)	1327 (99.18)	
Yes	52 (0.81)	28 (0.79)	13 (0.87)	11 (0.82)	
Usage					0.929
Intravenous infusion	4548 (71.10)	2531 (71.08)	1070 (71.43)	947 (70.78)	
Hypodermic injection	1849 (28.90)	1030 (28.92)	428 (28.57)	391 (29.22)	
Dosage (median [IQR])	380.00[162.00, 400.00]	384.00[162.00, 410.00]	380.00[162.00, 400.00]	360.00[162.00, 400.00]	0.377
Gender, n (%)					0.249
Male	1699 (26.56)	957 (26.87)	410 (6.13)	332 (24.81)	
Female	4698 (73.44)	2604 (73.13)	1088 (72.63)	1006 (75.19)	
Age (median [IQR])	66.00[59.00, 72.00]	66.00[59.00, 72.00]	65.00[59.00, 72.00]	66.00[59.00, 73.00]	0.674
Weight (median [IQR])	68.10[52.21, 85.08]	68.10[53.00, 85.30]	68.07[52.00, 85.00]	67.90[51.00, 85.28]	0.544
Leucopenia, n (%)					0.206
No	6237 (97.50)	3462 (97.22)	1469 (98.06)	1306 (97.61)	
Yes	160 (2.50)	99 (2.78)	29 (1.94)	32 (2.39)	

^*^Baseline differences between training and test sets arise from real-world data heterogeneity.

### Identification of predictive factors for leukopenia

**Figure 3:**
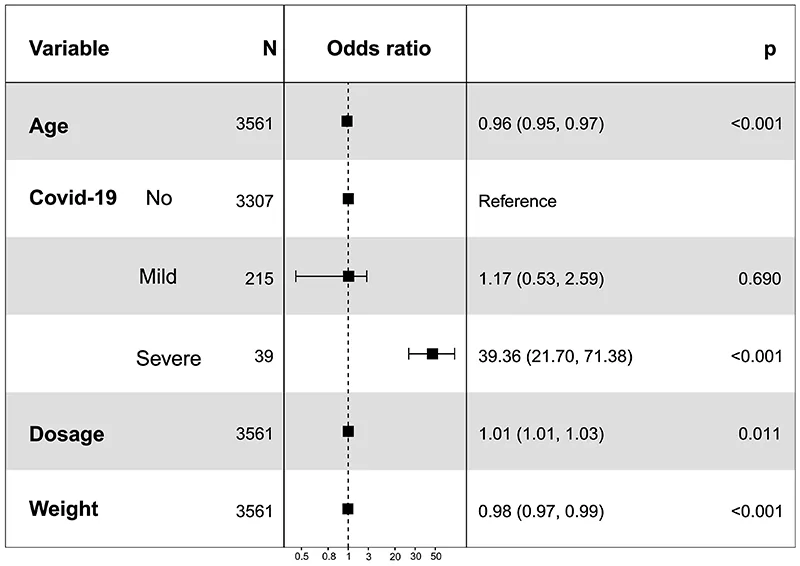
Forest plot of logistic regression analysis for the internal training set.

**Figure 4:**
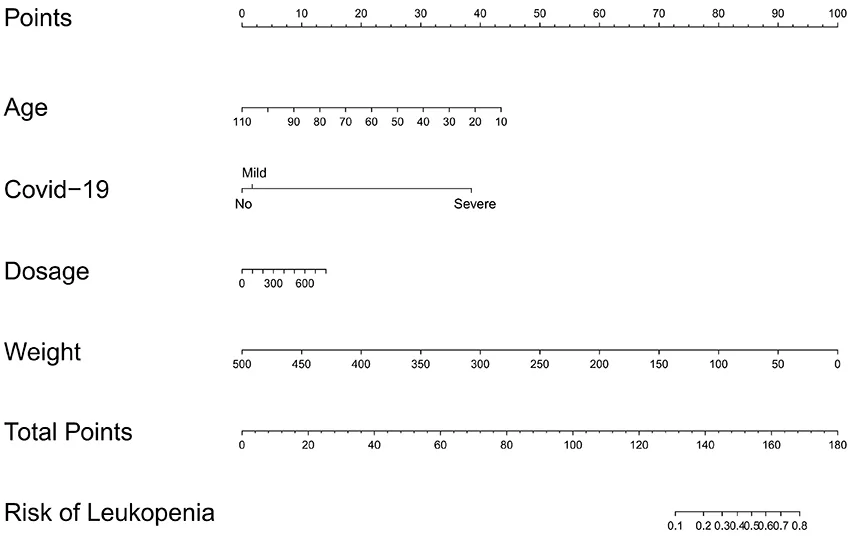
Nomogram for predicting the risk of leukopenia after tocilizumab treatment.

Using univariate and multivariate logistic regression analyses, we identified several independent predictors of leukopenia in patients treated with tocilizumab. In the multivariable model (Table 2), severe COVID-19 was strongly associated with leukopenia (OR: 39.36, 95%CI: 21.70-71.38, P<0.001), whereas mild COVID-19 did not show a statistically significant association (OR: 1.17, 95%CI: 0.53-2.59, P=0.690). Higher tocilizumab dosage remained a significant predictor (OR: 1.01, 95%CI: 1.01-1.03, P=0.011), while increasing age (OR: 0.96, 95%CI: 0.95-0.97, P<0.001) and higher body weight (OR: 0.98, 95%CI: 0.97-0.99, P<0.001) were associated with decreased odds of leukopenia. These four predictive factors were then analyzed using logistic regression in the internal training set (Figure 3) to create a prediction model. Finally, these predictive factors were integrated into a nomogram (Figure 4). Each predictor factor can be scored according to the points listed in the first row. The total score is the sum of each individual predictor's score. By adding the scores of all variables and locating the corresponding total score on the total score line, the probability of leukopenia after tocilizumab treatment occurrence can be estimated.

**Table 2:** Univariate and multivariate logistic regression analysis.

Characteristics	Univariate analysis			Multivariate analysis		
	OR	95%CI	P	OR	95%CI	P
Castleman's disease						
No	Ref					
Yes	2.33	0.93-5.83	0.07	NA	NA	NA
COVID-19						
No	Ref					
Mild	0.81	0.38-1.74	0.590	1.17	0.53-2.59	0.690
Severe	26.35	15.46-44.9	<0.001	39.36	21.7-71.38	<0.001
Giant cell arteritis						
No	Ref					
Yes	0.41	0.2-0.84	0.010	0.96	0.45-2.04	0.916
Juvenile idiopathic arthritis						
No	Ref					
Yes	0.08	0.01-1.37	0.980	NA	NA	NA
Rheumatoid arthritis						
No	Ref					
Yes	0.91	0.63-1.32	0.621	NA	NA	NA
Still's disease						
No	Ref					
Yes	1.66	0.6-4.57	0.33	NA	NA	NA
Takayasu's arteritis						
No	Ref					
Yes	1.57	0.38-6.49	0.542	NA	NA	NA
Usage						
Intravenous infusion	Ref					
Hypodermic injection	0.61	0.41-0.90	0.012	1.09	0.69-1.74	0.703
Dosage	1.01	1.01-1.06	<0.001	1.01	1.01-1.03	0.011
Gender						
Male	Ref					
Female	1.09	0.76-1.56	0.65	NA	NA	NA
Age	0.96	0.95-0.98	<0.001	0.96	0.95-0.97	<0.001
Weight	0.99	0.98-0.99	<0.001	0.98	0.97-0.99	<0.001

OR: Odds Ratio, CI: Confidence Interval; NA: not applicable.

### Calibration and verification of the nomogram prediction model

**Figure 5:**
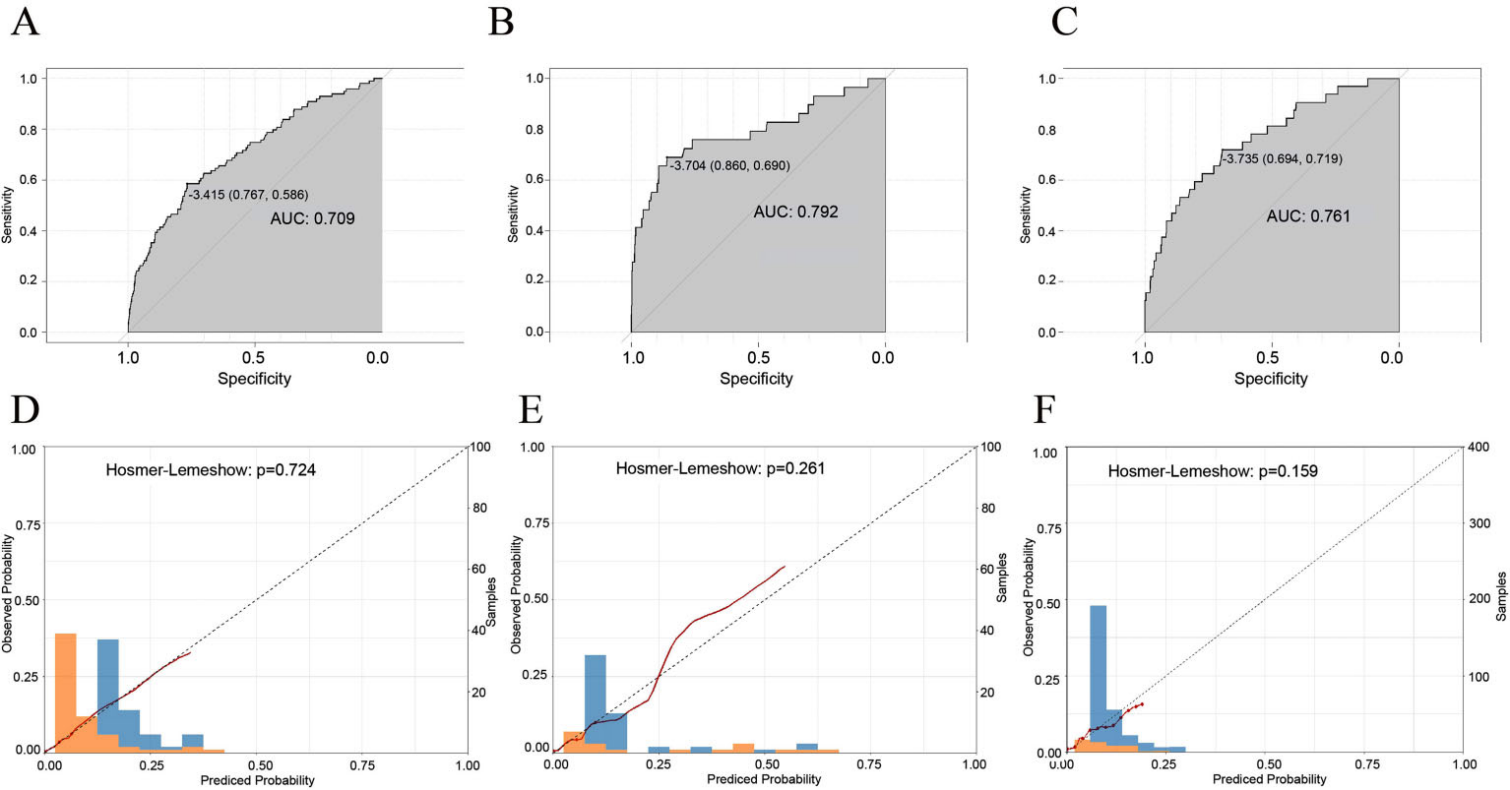
Receiver operating characteristic curves to evaluate the reliability of the prediction model and the calibration curves of the nomogram. **A** and **D**, internal training set; **B** and **E**, internal testing set; **C** and **F**, external testing set. AUC: area under the receiver operating characteristic curve.

**Figure 6:**
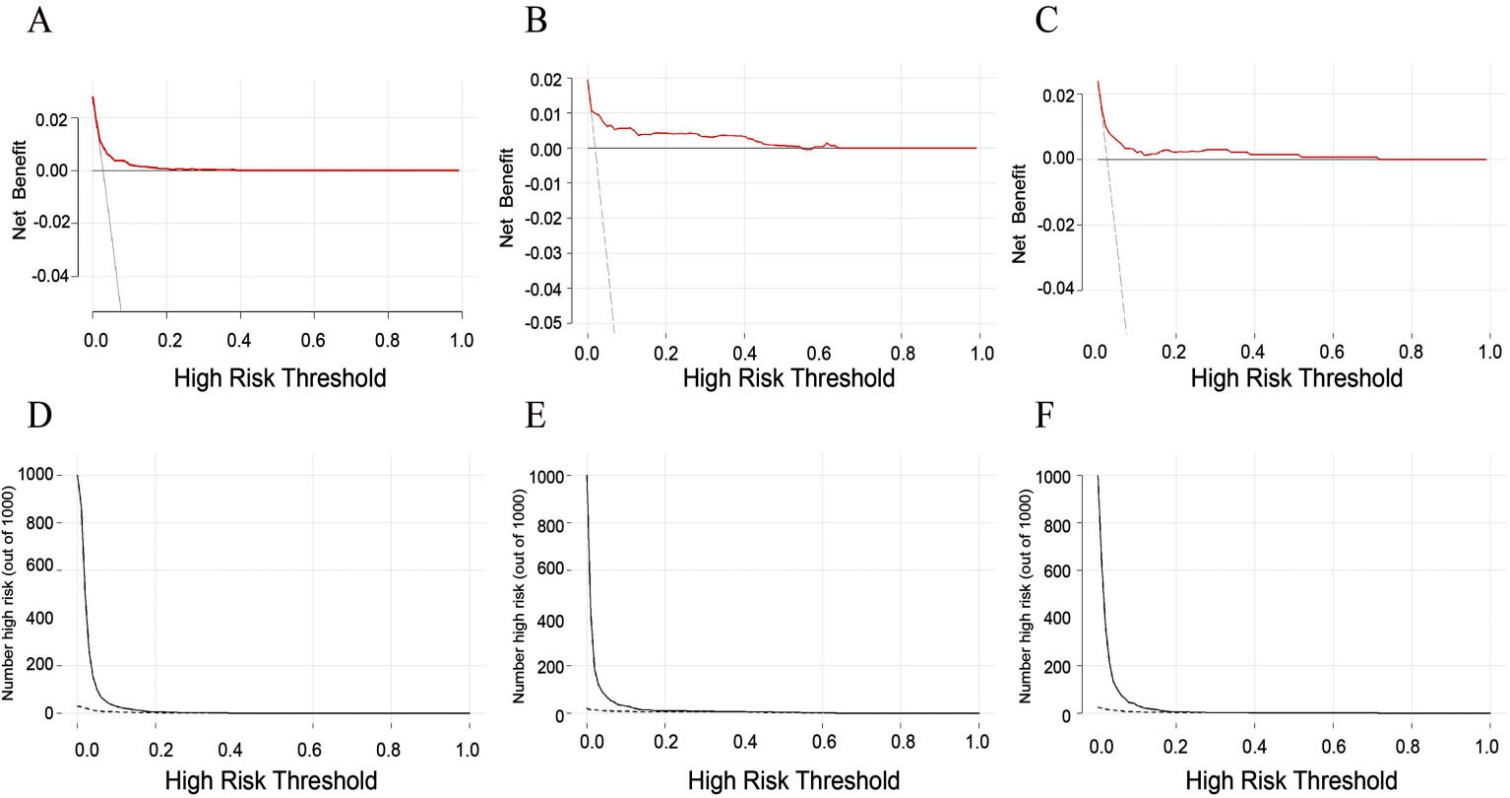
Decision curve analysis (DCA) and clinical impact curve analysis (CICA) for the nomogram model. **A**, DCA based on the internal training set; **B**, DCA based on the internal testing set; **C**, DCA based on the external testing set; **D**, CICA based on the internal training set; **E**, CICA based on the internal testing set; **F**, CICA based on the external testing set.

We evaluated the sensitivity and specificity of the prediction model using ROC curves. The ROC curve evaluates binary classification model performance by plotting the true positive rate (sensitivity) against the false positive rate (1-specificity), with the AUC quantifying overall discriminative ability (where 0.5 indicates random chance and 1.0 represents perfect discrimination). The AUC for the internal training set was 0.709. The model exhibited a sensitivity of 0.586 and a specificity of 0.767. For the internal testing set, the AUC was 0.792. Accordingly, the sensitivity and specificity were 0.690 and 0.860, respectively. In the external testing set, the AUC was 0.761. The model showed a sensitivity of 0.719 and a specificity of 0.694. These results demonstrate that the model we developed has high predictive accuracy (Figure 5A-C). To calibrate the prediction model, we created a calibration plot. Calibration curves compared predicted probabilities (x-axis) against observed event rates (y-axis), with the diagonal dashed line representing ideal prediction; closer alignment between the calibration curve and diagonal indicates better predictive accuracy. The P-values from the Hosmer-Lemeshow test for the internal training set, internal testing set, and external testing set were 0.724, 0.261, and 0.159, respectively. These results indicate no significant difference between the predicted and observed values, suggesting that the model has good calibration ability. The calibration curve results showed that the actual prediction curve closely aligns with the simulated prediction curve (Figure 5D-F). Additionally, DCA and CICA demonstrated that the nomogram provides higher net benefits in predicting the risk of leukopenia in patients receiving tocilizumab (Figure 6).

### Risk stratification based on the nomogram

**Figure 7:**
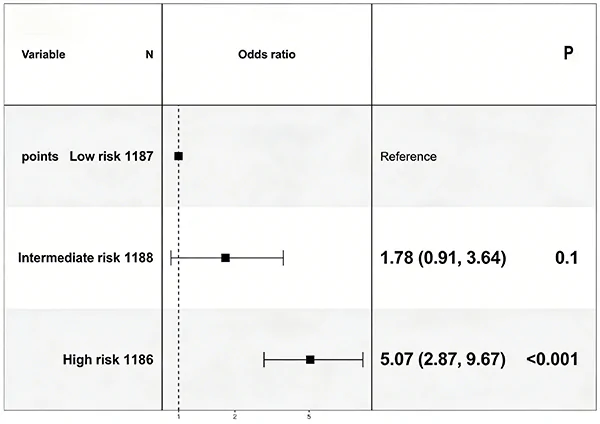
Stratified analysis of nomogram score and leukopenia risk.

To further investigate the correlation between the total score on the nomogram and the risk of leukopenia, we assigned scores to each independent factor included in the nomogram. We then calculated the total score for each patient based on the variable scores and divided the patients into three groups: low risk (57.03-130.91), intermediate risk (130.92-141.99), and high risk (142.00-219.80). Our analysis revealed significant differences in the risk of leukopenia between the groups. Compared with the low-risk group, patients classified as high risk by the nomogram had a substantially increased likelihood of developing leukopenia (OR: 5.07; 95%CI: 2.87-9.67; P<0.001). The intermediate-risk group showed a 1.78-fold higher odds (95%CI: 0.91-3.64), although this did not reach statistical significance (P=0.100) (Figure 7).

## Discussion

This study is the first to successfully develop and validate a clinical prediction model for leukopenia in adult patients receiving tocilizumab using data from the FAERS and Japanese PMDA databases. The model identifies several key predictive factors, including age, dosage, COVID-19 and weight, which are crucial in assessing the risk of leukopenia in these patients. The nomogram demonstrated good discriminative ability, effectively stratifying patients by leukopenia risk. After stratifying by COVID-19 severity, severe COVID-19 was strongly associated with leukopenia, whereas mild COVID-19 showed no significant association. The per-unit effects of dose, age, and weight remained numerically modest (dose per 1 mg: OR 1.01; age per 1 year: OR 0.96; weight per 1 kg: OR 0.98). It is important to emphasize that an OR close to 1.0 for a single unit does not imply clinical irrelevance: when expressed on clinically meaningful scales, these associations become appreciable. For example, a 10-year increase in age corresponds to an OR of ≈0.66 (0.96^10^≈0.66), indicating an approximately 34% lower odds of leukopenia; a 100-mg increase in dose corresponds to an OR of ≈2.70 (1.01^100^≈2.70), indicating a clinically meaningful increase in risk across typical dosing ranges; and a 10-kg increase in body weight corresponds to an OR of ≈0.82 (0.98^10^≈0.82). Thus, although the effects per single year, milligram, or kilogram appear small, their cumulative impact across realistic differences in age, dose, and weight can be substantial and should be considered in clinical decision-making.

In this study, age, dosage, weight, and COVID-19 were identified as significant predictive factors. We found that younger patients were more prone to leukopenia after receiving tocilizumab, which was inconsistent with previous assumptions (7,12). Research suggests that younger patients tend to have more active immune responses, potentially leading to stronger inflammatory reactions (13,14). When tocilizumab suppresses inflammation by blocking the IL-6 signaling pathway, it may cause greater inhibition of the hematopoietic system (15). Additionally, immune-related diseases in younger patients are often in a more active state at the time of diagnosis, with higher levels of inflammation (16,17). This elevated inflammatory activity may exacerbate bone marrow depletion and inhibit leukopoiesis. Although younger patients typically have better bone marrow reserve, these factors may make them more susceptible to leukopenia during tocilizumab treatment.

The immunosuppressive effect of tocilizumab is closely related to dose. The dose-dependent immunosuppressive effect of tocilizumab is mainly achieved by blocking IL-6 signaling pathway, and IL-6 itself is a key factor in maintaining granulocyte production and survival. Increasing the dose will exacerbate the inhibition of hematopoietic function, resulting in an increased risk of leukopenia. Tocilizumab blocks the biological effects of IL-6 by binding to the IL-6 receptor (18). As the dose increases, the occupancy of the receptor by the drug increases, leading to more complete inhibition of the signaling pathway. High doses can prolong the half-life of the drug and enhance the sustained inhibition of IL-6 signaling, thus intensifying the interference with bone marrow hematopoietic function. After high dose of tocilizumab blocked IL-6 signal, the activation of STAT3 decreased, resulting in a weakened effect of granulocyte colony-stimulating factor (G-CSF), which affected the differentiation and maturation of neutrophils (19). IL-6 inhibition may reduce hematopoietic supporting factors (such as SCF and GM-CSF) secreted by bone marrow stromal cells, further inhibiting granulocyte production.

Clinical pharmacokinetic studies of tocilizumab show that body weight significantly influences drug exposure, particularly for fixed-dose subcutaneous regimens: patients with higher body weight tend to have lower peak and trough concentrations of the drug compared to lighter individuals receiving the same fixed dose (20). Because leukopenia under tocilizumab is believed to result from suppression of IL-6 signaling, which normally contributes to neutrophil production, mobilization from the bone marrow, and survival, greater drug exposure (which may occur in lighter or lower-weight patients for a given dose) could lead to more pronounced decreases in white blood cell counts (4). Conversely, heavier patients receiving fixed doses may have relatively lower drug levels, potentially reducing the risk of leukopenia. Thus, weight acts as a modifier of exposure and may partially account for the observed association between higher body weight and reduced odds of leukopenia in multivariable analyses.

During tocilizumab treatment in COVID-19 patients, several hematological and hepatic adverse events have been observed, including thrombocytopenia, neutropenia, pruritus, and elevated liver enzymes (hepatotoxicity) (21). Among these, leukopenia represents one of the most frequently reported hematological adverse events (22). The synergistic interaction between COVID-19-induced inflammatory storm/immune exhaustion and tocilizumab-mediated IL-6 inhibition significantly elevates the risk of leukopenia through multiple mechanisms. In severe COVID-19 cases, markedly elevated IL-6 levels contribute to cytokine storm formation, while tocilizumab blockade of IL-6 receptors inhibits STAT3 pathway phosphorylation, consequently attenuating G-CSF effects and directly impairing neutrophil differentiation and maturation. Concurrently, IL-6 suppression reduces hematopoietic support factors (e.g., SCF, GM-CSF) secretion by bone marrow stromal cells, and SARS-CoV-2 infection itself may damage the bone marrow microenvironment either through direct viral invasion or inflammatory mediators (e.g., TNF-α), further suppressing hematopoietic stem cell proliferation (23). Additionally, concomitant therapies in COVID-19 management, including corticosteroids (which may synergistically suppress immune responses) and certain antiviral agents (which may prolong tocilizumab metabolism), could exacerbate myelosuppression (24). The immunocompromised state in critically ill patients (characterized by lymphopenia and monocyte dysfunction) impairs hematopoietic recovery, while sustained high-dose tocilizumab-mediated IL-6 signaling blockade may exceed hematopoietic tolerance thresholds, leading to significantly increased incidence of neutropenia (25). Early clinical observations documented a COVID-19-associated ARDS case showing clinical improvement post-tocilizumab therapy but subsequently developing severe persistent neutropenia (26). Another report described a Nepalese male COVID-19 patient who developed hematological crisis with severe neutropenia (ANC=300 cells/μL) and leukopenia, which was successfully managed through cautious tocilizumab and G-CSF combination therapy, achieving both clinical recovery and neutrophil count normalization (27). Avni et al. (28) demonstrated significantly higher neutropenia incidence (absolute neutrophil count (ANC)<500-1000 cells/μL) in tocilizumab-treated patients compared to controls. Notably, African American patients exhibit greater susceptibility to baseline ANC reduction (66.7% with ANC<2000 cells/μL), mechanistically linked to Duffy null phenotype (erythrocyte Duffy antigen deficiency) (29). A representative case showed an African American COVID-19 patient developing severe prolonged neutropenia (≥4 weeks duration, ANC decreasing from 9.8×10^9^/L to 0.52×10^9^/L post-tocilizumab) (30). These findings underscore the necessity for rigorous neutrophil monitoring and timely dose adjustment or treatment discontinuation to optimize the risk-benefit balance between anti-inflammatory efficacy and hematopoietic protection.

This study has several notable strengths. First, it leverages large-scale, real-world data from the FAERS and PMDA databases, which provide a robust foundation for developing predictive models in pharmacovigilance. The inclusion of an external validation cohort enhances the generalizability of the findings and underscores the model's applicability across diverse patient populations. Second, the use of advanced statistical techniques, including multivariable logistic regression, nomogram construction, and decision curve analysis, ensures a rigorous and clinically relevant approach to risk prediction. Third, the identification of COVID-19 as a significant predictor of leukopenia adds a novel dimension to the understanding of tocilizumab-related adverse events. These strengths collectively highlight the study's contribution to the field of precision medicine and its potential to improve patient outcomes.

Although the results are promising, it is important to acknowledge several limitations of this study. First, this study relies on data from the FAERS and PMDA databases, which are based on voluntarily reported adverse events. This method may introduce reporting bias, thus limiting the generalizability of our findings. While spontaneous reporting systems like FAERS are subject to underreporting and variable data quality, our use of standardized exclusion criteria, MedDRA coding, and external validation with PMDA data minimizes these limitations. The consistency of results across cohorts supports model robustness despite potential reporting biases. Second, despite performing an initial analysis, the generalizability of our results is still constrained by the potential limitations in sample size. Despite stratified sampling, minor covariate differences between training and test sets highlight the challenges of real-world data. Future studies may employ propensity score matching to further harmonize groups, though our external validation with PMDA data supports generalizability. Third, other unaccounted factors, such as underlying diseases, drug interactions, or genetic polymorphisms, could also significantly impact the model. Although we have identified tocilizumab as the primary suspected drug in the reports, it is important to acknowledge that the information on concomitant medications in the database is often incomplete and inconsistently reported. This limitation may affect our assessment of drug-drug interactions, particularly when evaluating the independent association between tocilizumab and leukopenia. For instance, methotrexate, a commonly used treatment for rheumatoid arthritis, is itself associated with leukopenia as a known side effect. Due to the incomplete documentation of concomitant medications in both the FAERS and PMDA databases, we were unable to systematically evaluate the impact of such drug interactions on the study findings. This limitation may introduce potential bias. Therefore, future research should focus on controlling these potential confounding factors to improve model accuracy. Fourth, due to individual variability, drug metabolism and clearance rates may be influenced by several factors, such as liver function and renal function. Finally, other respiratory infections (e.g., bacterial pneumonia, influenza) may also influence the risk of hematological adverse events during tocilizumab therapy through immune dysregulation or synergistic effects with IL-6 blockade. However, due to the absence of systematic data on respiratory comorbidities and concurrent infections in the FAERS and PMDA databases, we were unable to assess their impact in this study. Future studies should incorporate comprehensive respiratory disease data to further evaluate their role in tocilizumab-associated leukopenia. Additionally, the lack of dynamic monitoring data on tocilizumab drug concentrations in this study limits the in-depth analysis of the relationship between drug concentration and adverse reactions.

We acknowledge several important limitations related to the time frame of data collection and the inherent biases of spontaneous reporting systems. COVID-19 cases are concentrated in the latter part of our study period (from 2020 onward), and the pandemic altered clinical practice in multiple ways - including increased reporting frequency, more intensive laboratory monitoring, and changes in concomitant therapies - any of which could inflate the relative reporting rate of COVID-19-associated leukopenia. As the pandemic wanes and clinical practices evolve, the relative contribution of COVID-19 to predicted risk may change; therefore, the model's performance and calibration should be validated and, if necessary, recalibrated in independent, time-stratified datasets or prospective cohorts that reflect non-pandemic care. We also note limitations related to outcome definition. Although we used a prespecified laboratory threshold (WBC<4.0×10^9^/L) to define leukopenia for reproducibility, the events included in our analysis derive from clinician-reported adverse-event submissions and therefore often reflect clinical concern in addition to numeric abnormalities. Consequently, some clinically meaningful leukopenia events - documented by physicians despite WBC values marginally above the threshold because of symptoms, trends, or co-morbid infection - may not be fully captured by our definition. The structure and completeness of spontaneous reports limit our ability to identify these “sub-threshold but clinically significant” cases comprehensively. Future studies should therefore consider composite outcome definitions that integrate laboratory values, physician diagnosis, and clinical symptoms, and should seek prospective validation in cohorts with complete clinical and temporal data to better characterize the true clinical risk of tocilizumab-associated leukopenia.

## Conclusion

In conclusion, our study highlights the utility of large-scale real-world databases, such as FAERS and PMDA, in developing predictive models for drug-induced adverse events. By integrating these databases, we have provided a novel, clinically relevant tool to enhance the safety and efficacy of tocilizumab therapy. The nomogram offers a reliable method for identifying patients at high risk of leukopenia and could be a valuable asset in clinical practice. Further validation and prospective studies are needed to confirm its effectiveness across different patient populations and healthcare settings.

## Data Availability

The data that support the findings of this study are available in FAERS at https://www.fda.gov/. These data were derived from the following resources available in the public domain: FAERS, https://fis.fda.gov/sense/app/95239e26-e0be-42d9-a960-9a5f7f1c25ee/sheet/7a47a261-d58b-4203-a8aa-6d3021737452/state/analysis; PMDA, https://www.pmda.go.jp/english/.

## Supplementary Materials

Click to view [pdf].
