# Supplementary material for: Nomogram-based prediction model for tocilizumab-induced leukopenia in adults using FAERS and PMDA data
Source: Braz J Med Biol Res. 2026 Aug 21;59:e15395. doi: 10.1590/1414-431X2026e15395 (PMC13502765; doi:10.1590/1414-431X2026e15395)
Supplement: Supplementary file 1 [file 1414-431X-bjmbr-59-e15395-suppl.pdf]

Study Manual for Identification of Leukopenia Cases

Version: v1.0
Date: April 7, 2025

1. Purpose and Scope

This study manual describes, in detail, the procedures used to identify leukopenia and neutropenia cases from the FAERS and PMDA databases, including terminology mapping rules, duplicate-report adjudication, and quality-control and traceability requirements. The manual is intended for use by the study data-processing team and by reviewers or auditors.

2. Definitions and Terminology

2.1 Leukopenia:

For this study, case ascertainment prioritizes diagnostic terminology (PT/LLT) mapped to MedDRA Preferred Terms. Where available, laboratory results are used as a secondary criterion: peripheral white blood cell count (WBC) $<4.0\times10^9$/L is considered leukopenia. When neutropenia data are explicitly reported, absolute neutrophil count (ANC) $<1.5\times10^9$/L is recorded as a secondary indicator.

2.2 PT (Preferred Term) / LLT (Lowest Level Term):

MedDRA terminology levels used for coding adverse events. This study uses a single, standardized MedDRA dictionary (MedDRA® Version 26.1).

2.3 CASEID / PRIMARYID:

Database fields used to uniquely identify a report/case. These identifiers are primary keys for initial deduplication and provenance tracking.

2.4 Duplicate record:

A duplicate denotes multiple reports that refer to the same clinical event or the same patient within the same event time window, potentially submitted by different reporters or through different channels. Duplicate reports are merged to avoid bias in event counts and statistical analyses.

3. Data sources and study period

3.1 FAERS

We used the FDA Adverse Event Reporting System (FAERS) public releases for drugs and biologics covering the period from January 1, 2010 to September 30, 2024. All source files and their release versions were archived, and the date of data extraction and the original file names are recorded in our data log.

3.2 PMDA

We used the Pharmaceuticals and Medical Devices Agency (PMDA) adverse-event dataset for the same period (January 1, 2010 to September 30, 2024). The PMDA data export version and extraction metadata are documented; PMDA information is available at https://www.pmda.go.jp/english/.

4. Cohort selection

Reports mentioning tocilizumab were extracted from the raw FAERS and PMDA export files. We applied the following exclusion criteria: non-adult reports (when age information was available; only patients aged ≥18 years were retained), records with implausible body weight values (<15 or >150 kg), reports lacking sufficient information to confirm exposure to tocilizumab, and reports containing any evidence of viral infection. All selection steps, intermediate counts, and the final included sample size are provided in the study flowchart and in the data-processing log to ensure reproducibility.

5. MedDRA PT list (all included Preferred Terms)

Complete list: *Leukopenia*; *Neutropenia*; *Agranulocytosis*; *Decreased white blood cell count*; *Neutrophil count decreased*; *Leukocytopenia*; *Granulocytopenia*.

6. Mapping procedure and rules

• **Independent review.** Two independent reviewers (L.J.W. and X.C.Z.) screened all extracted reports and completed a standardized "PT mapping form". Each reviewer classified every PT/LLT listed in a report as *related to leukopenia* (Yes), *not related* (No), or *uncertain*, and recorded the rationale for their decision and whether the determination was based on reported laboratory values. Reviewers performed their assessments independently and did not share results during the initial mapping to preserve blinding.

• **Adjudication.** Discrepant classifications were submitted to a third adjudicator (X.L.B.) for final resolution. The adjudicator recorded the final decision and the reasoning; all mapping decisions and adjudication notes are retained in an adjudication log.

• **Decision rules.** The following operational rules were applied when assigning events:

– Reports containing the term *neutropenia* or *agranulocytosis* were classified as neutropenic events.

– Reports containing only *decreased white blood cell count* were evaluated for supporting information (reported ANC/WBC values or explicit clinician judgment); such reports were counted as leukopenia only if the supporting information was present.

– If a single report included multiple PTs, the most specific or clinically severe PT was used as the primary classification; however, all related PTs were retained in the database for secondary analyses.

7. Deduplication procedure

• **Automated matching stage:** Records were first grouped by CASEID/PRIMARYID, when available. For records without a common unique identifier, we performed multi-field deterministic matching using event onset date, age, sex, country/region, and drug exposure dates (among other key fields).

• **Retention rules:** When records were judged to represent the same clinical event, we retained the report with the most complete information and the most recent FDA received date. If FDA received dates were not comparable or unavailable, we retained the record containing the most detailed clinical narrative or laboratory data.

• **Manual adjudication stage:** Suspected duplicates that could not be resolved by

automated matching (for example, records with different CASEID values but highly similar key fields) were reviewed independently by two reviewers. Each reviewer documented the rationale for their decision; disagreements were resolved by a third adjudicator.

- **Result consolidation:** All adjudication outcomes were merged into a final adjudication table. Records classified as leukopenia were flagged with outcome_flag=1; records not meeting the leukopenia definition were flagged with outcome_flag=0. All provenance information and merge rationales are retained in the deduplication log to ensure traceability.

8. Definition and Stratification of COVID-19 Severity

To assess the potential impact of COVID-19 severity on tocilizumab-associated leukopenia, we performed a stratified analysis classifying patients into three groups: no COVID-19, mild COVID-19, and severe COVID-19. Because FAERS and PMDA are spontaneous reporting systems that do not systematically capture standardized clinical severity stages, we developed and applied a prespecified mapping algorithm to infer COVID-19 severity from the available structured data fields and narrative information. COVID-19 cases were first identified by searching the 'Indication' and 'Reason for Use' fields, as well as verbatim reporting text, for terms indicative of SARS-CoV-2 infection (e.g., 'COVID-19', 'SARS-CoV-2', 'coronavirus infection'). For each identified case, severity was then determined using the following hierarchical approach based on the 'seriousness' fields, MedDRA Preferred Terms, and keywords:

- **Severe COVID-19**: Reports that met any of the following criteria were classified as severe: 1) a 'seriousness' designation indicating a life-threatening event, hospitalization (initial or prolonged), disability, or death; 2) presence of PTs or keywords referring to intensive care unit (ICU) admission, mechanical ventilation, or acute respiratory distress syndrome (ARDS); or 3) explicit mention of high-flow oxygen, non-invasive or invasive ventilation.
- **Mild COVID-19**: Reports documenting a COVID-19 diagnosis but lacking any of the severe criteria listed above. These included cases where hospitalization was either not reported or, if present, did not involve ICU admission, life-threatening conditions, or advanced respiratory support.
- **No COVID-19**: All remaining reports without evidence of COVID-19 infection.

9. All positive Preferred Terms (PT) associated with Tocilizumab.

System Organ Class	PT code	PT
Blood and lymphatic system disorders	10029354	Neutropenia
	10001507	Agranulocytosis
	10018687	Granulocytopenia
	10029354	Neutropenia
	10051645	Idiopathic neutropenia
	10055128	Autoimmune neutropenia
	10024384	Leukopenia
